# Genome-Wide Association Studies of Free Amino Acid Levels by Six Multi-Locus Models in Bread Wheat

**DOI:** 10.3389/fpls.2018.01196

**Published:** 2018-08-14

**Authors:** Yanchun Peng, Hongbo Liu, Jie Chen, Taotao Shi, Chi Zhang, Dongfa Sun, Zhonghu He, Yuanfeng Hao, Wei Chen

**Affiliations:** ^1^College of Plant Science and Technology, Huazhong Agricultural University, Wuhan, China; ^2^National Key Laboratory of Crop Genetic Improvement, National Center of Plant Gene Research, Huazhong Agricultural University, Wuhan, China; ^3^School of Chemical Science and Engineering, Royal Institute of Technology, Stockholm, Sweden; ^4^Institute of Crop Science, National Wheat Improvement Center, Chinese Academy of Agricultural Sciences, Beijing, China

**Keywords:** wheat, free amino acid (FAA), genome-wide association studies, multi-locus models, QTNs

## Abstract

Genome-wide association studies (GWAS) have been widely used to dissect the complex biosynthetic processes of plant metabolome. Most studies have used single-locus GWAS approaches, such as mixed linear model (MLM), and little is known about more efficient algorithms to implement multi-locus GWAS. Here, we report a comprehensive GWAS of 20 free amino acid (FAA) levels in kernels of bread wheat (*Triticum* *aestivum* L.) based on 14,646 SNPs by six multi-locus models (FASTmrEMMA, FASTmrMLM, ISISEM-BLASSO, mrMLM, pKWmEB, and pLARmEB). Our results showed that 328 significant quantitative trait nucleotides (QTNs) were identified in total (38, 8, 92, 45, 117, and 28, respectively, for the above six models). Among them, 66 were repeatedly detected by more than two models, and 155 QTNs appeared only in one model, indicating the reliability and complementarity of these models. We also found that the number of significant QTNs for different FAAs varied from 8 to 41, which revealed the complexity of the genetic regulation of metabolism, and further demonstrated the necessity of the multi-locus GWAS. Around these significant QTNs, 15 candidate genes were found to be involved in FAA biosynthesis, and one candidate gene (*TraesCS1D01G052500*, annotated as tryptophan decarboxylase) was functionally identified to influence the content of tryptamine *in vitro*. Our study demonstrated the power and efficiency of multi-locus GWAS models in crop metabolome research and provided new insights into understanding FAA biosynthesis in wheat.

## Introduction

Genome-wide association studies (GWAS) have largely been applied to the genetic dissection of complex traits in plants. With the landmark GWAS study of 107 phenotypes in *Arabidopsis* (Atwell et al., 2010), numerous other studies have been successfully performed, including those addressing the flowering time and grain yield in rice (Huang et al., 2012; Yang W. et al., 2014), salinity tolerance in barley (Fan et al., 2016), male inflorescence size in maize (Wu et al., 2016), floret fertility in wheat (Guo et al., 2017), and the reducing levels of cucurbitacin in cucumber domestication (Shang et al., 2014). Of these studies, the mixed linear model (MLM) has been adopted most frequently owing to its effective control of spurious associations (Yu et al., 2006). However, as a single-locus GWAS approach, MLM leads to missing some significant loci because of the conservative Bonferroni correction (0.05/*m_e_*, where *m_e_* is the number of effective markers) and the stringent criterion of the significance test (Wang et al., 2016). To address this issue, several multi-locus models have been developed, such as Bayesian LASSO (Hoggart et al., 2008), ISISEM-BLASSO (Tamba et al., 2017), pLARmEB (Zhang et al., 2017), and pKWmEB (Ren et al., 2018). Because of the multi-locus nature, the obvious superiority of these approaches is that no Bonferroni correction is demanded, hence, a looser significance criterion can be adopted, and more-powerful quantitative trait nucleotides (QTNs) can be detected (Wang et al., 2016).

Plants produce a vast array of metabolites that provide nutrition and medicine for humans (Saito and Matsuda, 2010; Chae et al., 2014). Unraveling the diversity of the plant metabolome and its underlying mechanism has attracted increasing research interest in the past decade (Schwab, 2003; De Luca et al., 2012). Recent research showed that GWAS coupled with metabolome analysis (mGWAS) exhibited great potential to dissect the genetic and biochemical bases of metabolome diversity (Chan et al., 2011; Chen et al., 2014; Wen et al., 2014). Similar to complex traits such as plant height and grain weight, which are usually controlled by several loci with small effects (Huang et al., 2010; Yang W. et al., 2014), the production of plant metabolites is often controlled by pathways composed of multiple genes. For instance, levels of primary metabolites, such as amino acids, fatty acids and saccharides, tend to be controlled by small effects loci (Angelovici et al., 2013; Matsuda et al., 2015). Whereas, in contrast to primary metabolites, the contents of secondary metabolites are always controlled not only by a few major loci with large effects but also by additional numerous loci with small effects (Chan et al., 2010; Riedelsheimer et al., 2012). Although the single-locus mGWAS models have succeeded in identifying a number of genetic variants associated with thousands of metabolites, this methodology ignores the joint effects of multiple genetic markers on metabolites (Chan et al., 2010; Tamba et al., 2017). Therefore, multi-locus models are a valuable alternative method for mGWAS analysis.

Bread wheat or common wheat (*Triticum* *aestivum* L.) is one of the most important crops worldwide and provides approximately 20% of the energy, protein and dietary fiber consumed for human (Ling et al., 2013). The improvement of kernel quality has been a major target in breeding for a long time (Nelson et al., 2006; Jin et al., 2016). Although the seed amino acids are mainly present as components of storage proteins, free amino acids (FAAs) can contribute significantly to be the contents of limited essential amino acids in wheat kernels (Angelovici et al., 2013). To improve the amino acid compositions, both traditional plant breeding techniques and new biotechnologies can be utilized (Fernie and Schauer, 2009). Recently, with the rapid development of the next-generation sequencing technologies, some key genes influencing FAA concentrations have been identified in rice (Chen et al., 2016), maize (Deng et al., 2017), and *Arabidopsis* (Angelovici et al., 2013) via mGWAS, which showed great potential to accelerate breeding for balanced AA compositions. However, to our knowledge, no studies of dissecting genetic associations with FAA levels in wheat have been reported.

Here, to understand the genetic bases underlying the natural variation and the biosynthesis of FAAs in wheat kernels, we detected the levels of 20 FAAs with an LC-MS platform (Chen et al., 2013) from a highly diverse association panel of 182 accessions. We identified 328 significant QTNs (LOD > 3.0) with six multi-locus mGWAS models and assigned 15 candidate genes involved in FAA biosynthesis. As a proof of concept, we functionally identified *TraesCS1D01G052500* *in vitro*. Our study proved the efficiency of multi-locus GWAS models in metabolome research and provided new insights into understanding of FAA biosynthesis in wheat, which may facilitate metabolomics-based breeding for quality improvement.

## Materials and Methods

### Plant Material

A highly diverse association panel of 182 *Triticum* *aestivum* L. accessions, including both landraces and elite varieties (**Supplementary Table S1**), was described as before (Liu J. et al., 2017). All accessions were grown at Gaoyi in Hebei province and Dezhou in Shandong province during the 2016–2017 cropping season. Field trials were conducted in randomized complete blocks with three replicates at each location. Each plot contained three 2 m rows spaced 20 cm apart. Field trials followed standard agronomic wheat management practice. Ten mature seeds were randomly collected and pooled for metabolic profiling analysis.

### Genotyping

Total genomic DNA was extracted from young leaves for SNP arrays. The 182 accessions were genotyped using the Illumina wheat 90 K SNP by Capital Bio Corporation, Beijing, China^1^. Accuracy of SNP clustering was validated visually step by step. Of the 81,587 SNPs, those with minor allele frequencies (MAFs) < 0.05 and missing data >20% were excluded from further analysis (Liu J. et al., 2017) to avoid spurious MTAs, finally, a total of 14,646 SNPs were employed in the association panel for GWAS analysis (Dong et al., 2016). The physical positions of SNPs were obtained from the International Wheat Genome Sequencing Consortium website (IWGSC)^2^.

### Determination of AA Levels

A widely targeted metabolomic platform was applied to quantify the FAA contents in mature wheat kernel samples as described previously (Chen et al., 2013). The dried kernels were crushed using a mixer mill (MM 400, Retsch) for 1.2 min at 29 Hz. Then, 100 mg powder was weighted and extracted for 8 h at 4°C with 1.0 ml 70% aqueous methanol containing 0.1 mg/l lidocaine (internal standard). Extracts were centrifuged at 10,000 *g* for 10 min, and filtrated (SCAA-104, 0.22 μm pore size; ANPEL, Shanghai, China^3^ before LC–MS analysis. The HPLC conditions as follow: column, shim-pack VP-ODS C18; solvent system, water with 0.04% acetic acid and acetonitrile with 0.04% acetic acid; gradient program, 0 min, 100:0 V/V, 20.0 min, 5:95 V/V, 22.0, 5:95 V/V, 22.1, 95:5 V/V, 25.0, 95:5 V/V; flow rate, 0.25 ml min^−1^; temperature, 40°C; Injection volume, 5 μl. The MS parameters as follow: ion spray voltage (IS) 5,500 V; source temperature 500°C; ion source gas I (GSI), gas II (GSII), curtain gas (CUR) were set at 55, 60, and 25.0 psi, respectively, the collision gas (CAD) was high. A specific set of MRM (multiple reaction monitoring) transitions were monitored for each FAA (**Supplementary Table S2**), each MRM transition was obtained with a 5 ms pause time and 5 ms Dwell time, data were processed by Analyst 1.5.1 software, peak areas were integrated using a IntelliQuan algorithm. Endogenous concentrations of FAAs were quantified by calculating the peak area in comparison to standard curves obtained from authenticated standards (purchased from Sigma-Aldrich). Calibration curves were drawn by plotting at least four different concentrations of each FAA standard according to the peak area (Dong et al., 2014). Finally, to eliminate environmental effects, BLUPs (best linear unbiased predictor) across two environments were used as the phenotypic values for all subsequent analyses (Liu J. et al., 2017).

### GWAS Mapping

Free amino acid levels were simultaneously studied with a single-locus GWAS model (MLM) and six multi-locus GWAS models. The single-locus model was implemented by FaST-LMM program (Lippert et al., 2011), while multi-locus models were implemented by mrMLM (Wang et al., 2016), FASTmrMLM (Tamba, 2017), FASTmrEMMA (Wen et al., 2017), ISISEM-BLASSO (Tamba et al., 2017), pLARmEB (Zhang et al., 2017), and pKWmEB (Ren et al., 2018). The critical threshold for significantly associated SNPs was set at *LOD* > 3.0 for the six multi-locus models, and *P* = 0.05/14,646 = 3.41 × 10^−6^ (or -log _10_P -value = 5.5, Bonferroni correction) for MLM.

### Statistical Analysis

We used s/ȳ × 100 to calculate the values of coefficient variation (CV, %) for each FAA, where *s* and ȳ are the standard deviation (SD) and the mean of each FAA in the population, respectively. Spearman’s rank correlation coefficient was used to calculate the correlation between each pair of FAAs, and statistical significance was obtained by using Student’s *t*-test.

### *In vitro* Validation of Candidate Genes

Full-length cDNA of *TraesCS1D01G052500* was amplified with the primer using cDNA from Huaimai20 as a template. Clones were digested with *Bam*H I/*Eco*R I and directionally ligated to the pre-digested *pGEX-6p-1* vector. Error-free recombinant proteins were expressed in BL-21 (DE3) competent cells after induced by adding 0.1 mM isopropyl β-D-1-thiogalactopyranoside (IPTG) and growing continually for 12 h at 16°C. Cells were harvested and suspended in the lysis buffer [contains 500 mM NaCl, 50 mM Tris-HCl (pH 8.0), 10% glycerol, 5 mM β-mercaptoethanol and 1 mM PMSF] and lysed by high pressure. The crude extract was collected and clarified by centrifugation at 14,000 *g* for 1 h at 4°C, and the supernatant was stored at −80°C for future experiments.

The standard *in vitro* enzyme assay for the role of TraesCS1D01G052500 (tryptophan as substrate) was performed in a total volume of 20 μl containing 100 ppm PLP and 50 μM substrate in 50 mM Tris-HCl buffer (pH 8.0). After incubating at 37°C for 30 min, the reaction was stopped by adding 60 μl of methanol. The reaction mixture was then filtered through a 0.2 μm filter (Millipore) before being used for LC-MS analysis.

### Phylogenetic Analysis of Different Gene Families

We use the CLUSTALW (version 1.83) program to align the amino acid sequences and construct the neighbor-joining tree by MEGA5. Bootstrap values from 1,000 times are indicated at each node. Bar = 0.1 amino acid substitutions per site.

### Enzyme Kinetics

To determine the kinetic difference between TraesCS1D01G052500 and its homologs in rice (OsTDC1 and OsTDC3), their activities were measured using 50 ng of purified protein expressed from *E. coli*, with 10–1,250 μM different tryptophan (Sigma) as substrates and a fixed concentration of 50 ppm PLP (Sigma) as co-factor. The kinetic parameters were calculated using Michaelis–Menten model (SigmaPlot software, version 14.0). All reactions were run in duplicate and repeated twice.

## Results

### Natural Variation of Free Amino Acids in Wheat Kernel

**FIGURE 1:**
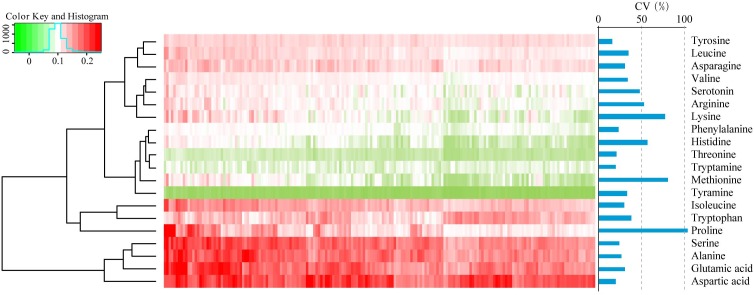
Hierarchical cluster analysis (HCA) and the coefficient variation (CV, %) of the levels of FAAs in 182 wheat accessions. Each accession is visualized in a single column, and each FAA is represented by a single row. Red indicates high level, whereas low FAA contents are shown in green.

To assess the phenotypic variation for FAAs in dry, mature wheat kernels, the absolute levels of 20 FAAs (alanine, arginine, asparagine, aspartic acid, glutamic acid, histidine, isoleucine, leucine, lysine, methionine, phenylalanine, proline, serine, serotonin, threonine, tryptamine, tryptophan, tyramine, tyrosine, and valine in nmol/mg dry wheat kernels) were quantified using LC-MS/MS as previously described (Chen et al., 2013). Visualization of the FAA profiling was performed by hierarchical cluster analysis (HCA), and accumulation of FAAs displayed a distinct phenotypic variation according to their abundance (**Figure 1**). Aspartic acid, glutamic acid, alanine and serine were the most highly abundant FAAs, with average concentrations of 0.37, 0.31, 0.30, 0.30 nmol/mg, respectively, while tyramine, threonine, and tryptamine were the less abundant, with average concentrations of 0.005, 0.02, 0.03 nmol/mg, respectively (**Supplementary Table S2**). The content of each FAA varied widely within the association panel, with variation ranging from a 2.30-fold difference in tyrosine to a 30.36-fold difference in proline and with the genetic coefficient variation (CV, %) ranging from 15.9 to 103.2, respectively (**Figure 1** and **Supplementary Table S2**). The relationships between 20 FAA values were evaluated by Spearman’s rank correlation, and strong positive correlations were identified between most of these FAAs, with the exceptions of tryptamine and tryptophan (**Supplementary Table S3**).

### Associated Loci Mapped by Different Models

To dissect the genetic basis of natural variation for FAA levels in mature wheat kernels, GWAS was performed using seven different models simultaneously. In total, 328 significant QTNs were identified by six multi-locus models (FASTmrEMMA, FASTmrMLM, ISISEM-BLASSO, mrMLM, pKWmEB, and pLARmEB) at a critical threshold of *LOD* > 3.0 (**Supplementary Table S4**), and the numbers of QTNs for the above six models were 38, 8, 92, 45, 117, and 28 (**Table 1**), respectively. Of these QTNs, 66 were detected by at least two different models; some QTNs, such as the association between lysine and SNP BS00003585_51 on chromosome 2B (747,603,047 bp), were simultaneously mapped by five different models (**Supplementary Table S4**). Only four significant SNP-trait associations were identified by the single-locus model (MLM) (**Table 1**), and could be also detected by some multi-locus models. Although 18 FAAs were found by FASTmrEMMA to be significantly associated with QTNs, the total number of QTNs is only 38, with an average of 2.1 QTNs per FAA. Comparatively, for the pKWmEB and ISISEM-BLASSO models, the average QTNs per trait reached 6.2 and 4.6, respectively (**Table 1**). The phenotypic variation explained by different loci varied from 0.1% (tyramine in pKWmEB) to 21.4% (aspartic acid in mrMLM), with an average of 5.6%. We also found that the same QTN shows different effects to explain the phenotypic variation in different models; for instance, the association between arginine and SNP BS00022811_51 on chromosome 7A (709,639,589 bp) with the *r*^2^ ranged from 0.1% in FASTmrEMMA to 19.7% in pKWmEB (**Supplementary Table S4**).

**Table 1:** Summary of significant QTNs identified by different models.

Model	FASTmrEMMA	FASTmrMLM	ISISEM-BLASSO	mrMLM	pKWmEB	pLARmEB	MLM
Number of traits with significant QTNs	18	6	20	15	19	11	4
Number of QTNs	38	8	92	45	117	28	4
Average QTNs per trait	2.1	1.3	4.6	3.0	6.2	2.5	1

**FIGURE 2:**
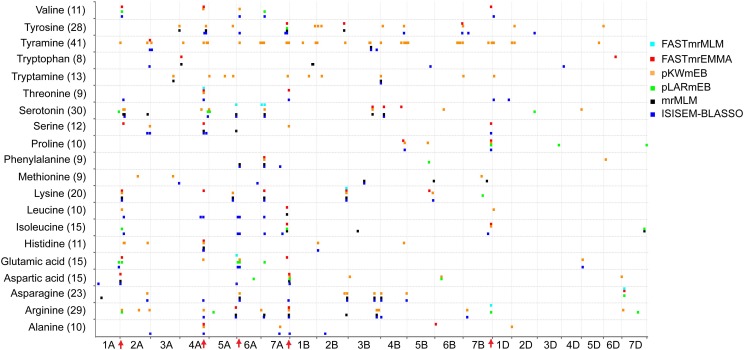
Chromosomal distribution of QTNs identified in this study. The *x*-axis indicates genomic locations by chromosomal order, and the significant QTNs are plotted against genome location. Each row represents one QTN identified by a different model. The red arrows show the QTN hotspots.

The number of significant QTNs also varied widely among different FAAs, ranging from 8 for tryptophan to 41 for tyramine (**Figure 2**), indicating the complex genetic regulation of FAAs. The chromosomal distribution of all identified QTNs revealed that A genome had the greatest number of significant associations, while only few QTNs were detected in the D genome (**Figure 2**). Since QTNs were not distributed evenly on the chromosomes (Deng et al., 2017), five QTN hotspots were observed on chromosomes 2A, 4A, 6A, 7A, and 7B, with the most obvious one being that more than 18 QTNs can be detected between 7 FAAs and SNP RAC875_c1022_3059 (located at 595,984,457 bp on chromosome 4A) (**Figure 2** and **Supplementary Table S4**). The candidate genes underlying these QTN hotspots could include transcriptional factors, transporters or some other rate-limiting enzymes of the amino acid metabolic pathway.

### Candidate Genes Underlying QTNs

Notably, the 328 significantly QTNs facilitated the assignment of candidate genes. To identify them, the flanking sequences corresponding to the SNP markers significantly associated with FAA levels were used in BLASTx search against NCBI database^4^. In most cases, the chemical structure combining with the existing knowledge of the biosynthetic pathway of the amino acids allowed the tentative assignment of a protein sequence that is biochemically related to the associated FAAs. Notably, 15 candidate genes involved in FAAs anabolism or catabolism were identified by mGWAS in this study (**Table 2**), based on the wheat reference genome information (see footnote 2).

**Table 2:** Summary of 15 candidate genes significantly associated with FAA levels.

Traits	Chr	Lead SNP position (bp)	*r*^2^ (%)^a^	LOD	Candidate gene^b^	Annotation
Glutamic acid	1A	558,490,011	6.9	3.5	TraesCS1A01G390300	Glutamate receptor
Alanine	4A	595,984,457	15.6	5.7	TraesCS4A01G294100	Aminopeptidase
Asparagine	4B	14,124,082	6.1	4.1	TraesCS4B01G020000	Aminopeptidase
Tryptamine	1D	34,621,416	6.7	3.8	TraesCS1D01G052500	Tryptophan decarboxylase
Tyramine	3B	543,718,678	1.9	5.5	TraesCS3B01G340000	Tyrosine decarboxylase
Glutamic acid	5D	31,273,563	10.1	4.2	TraesCS5D01G031800	Amino acid transporter
Isoleucine	7A	660,464,837	15.9	5.7	TraesCS7A01G464900	Amino acid transporter
Tyrosine	2B	689,871,912	8.3	4.5	TraesCS2B01G493000	Amino acid permease
Arginine	7B	105,558,975	4.4	3.9	TraesCS7B01G093200	Amino acid permease
Methionine	3B	408,354,812	14.4	4.3	TraesCS3B01G253600	Amino acid transporter
Valine	4A	593,337,515	9.6	3.6	TraesCS4A01G287900	Peptide transporter
Tyramine	3B	582,466,573	17.0	4.9	TraesCS3B01G369800	Aminotransferase
Lysine	2A	41,237,242	9.2	4.5	TraesCS2A01G088600	Pyruvate decarboxylase
Histidine	2B	26,581,220	16.1	7.3	TraesCS2B01G053600	Pyruvate dehydrogenase
Lysine	2B	747,603,047	9.4	5.8	TraesCS2B01G553300	Shikimate kinase

*^a^The phenotypic variance explained by the corresponding locus. ^b^A possible biological candidate gene in the locus or the nearest annotated gene to the lead SNP. More information is listed in **Supplementary Table S4**.*

**FIGURE 3:**
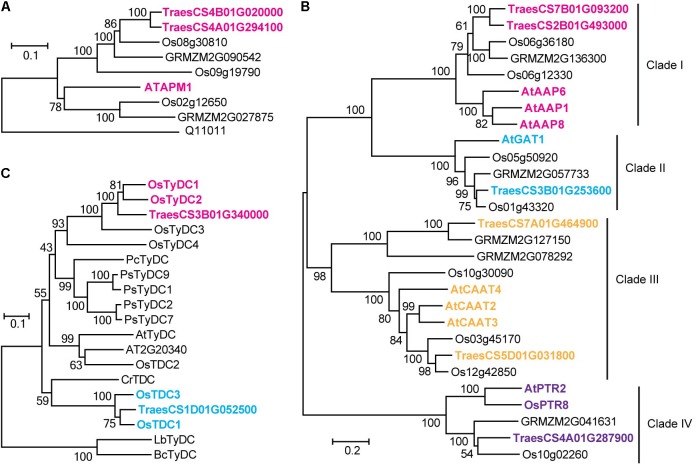
Homologous amino acid sequences of aminopeptidase gene family **(A)**, tyrosine decarboxylase and tryptophan decarboxylase gene families **(B)**, and amino acid permease, amino acid transporter and peptide transporter gene families **(C)** from multiple species were collected and aligned. The neighbor-joining trees were constructed using MEGA software and tested using bootstrap method at replication number of 1000. Phylogenetic analysis of different gene families assigned in the study. Os, *Oryza sativa*; At, *Arabidopsis thaliana*; Pc, *Petroselinum crispum*; Ps, *Papaver somniferum*; Cr, *Catharanthus roseus*; Lb, *Lactobacillus brevis*; Bc, *Bacillus cereus*.

A significant QTN between the levels of glutamic acid and the SNP Excalibur_c35310_375 was identified on chromosome 1A; this SNP is located 0.5 Mb away from *TraesCS1A01G390300* (encoding a putative glutamate receptor). The high homology (58% identity at amino acid level) between *TraesCS1A01G390300* and the glutamate receptor gene *AtGLR3.5* (Teardo et al., 2015) suggests that *TraesCS1A01G390300* is likely the candidate gene underlying this locus. The SNP RAC875_c1022_3059 was significantly associated with 7 FAAs (**Supplementary Table S4**), which is comprised a hotspot on chromosome 4A as mentioned above. The high sequence identity (61% at the amino acid level) between adjacently located gene *TraesCS4A01G294100* (0.4 Mb to SNP RAC875_c1022_3059) and *AtAPM1* (Murphy et al., 2002), an aminopeptidase in *Arabidopsis*, suggests that *TraesCS4A01G294100* is likely the candidate gene underlying this QTN. Similarly, *TraesCS4B01G020000* (also encoding a putative aminopeptidase), was assigned as the candidate gene underlying the content of asparagine. The associations were further supported by phylogenetic analysis (**Figure 3A**).

Levels of tryptamine were significantly associated (LOD = 3.8) with the SNP BS00012936_51 on chromosome 1D that is 1.0 Mb away from *TraesCS1D01G052500*, which encodes a protein annotated as tryptophan decarboxylase, suggesting that TraesCS1D01G052500 catalyzes the key step of tryptamine biosynthesis. Similarly, *TraesCS3B01G340000* (encoding a putative tyrosine decarboxylase) was assigned as the candidate gene underlying the levels of tyramine. The high sequence identities between *TraesCS1D01G052500* and *OsTDC1* (88% at the amino acid level, Kanjanaphachoat et al., 2012), *TraesCS3B01G340000* and *OsTyDC2* (79% at the amino acid level, Kang et al., 2007) further supported the realness of these QTNs (**Figure 3B**).

Six candidate genes putatively annotated as amino acid transporters (AATs) or amino acid permeases (AAPs) were identified by mGWAS (**Table 2**). We investigated the phylogenetic relationships among the AATs (or AAPs) by constructing the phylogenetic tree with a neighbor-joining algorithm based on the amino acid sequences of these candidate genes and a collection of nine reported genes (Dietrich et al., 2004; Hirner et al., 2006; Meyer et al., 2006; Lee et al., 2007; Yang H. et al., 2014; Santiago and Tegeder, 2016). As a result, characterized AATs (or AAPs) were sorted into four major clades (**Figure 3C**). Closer examination of the phylogeny in clade III reveled that *TraesCS5D01G031800* lies next to *AtCAAT2*, *AtCAAT3*, and *AtCAAT4*, three cationic amino acid transporters from *Arabidopsis* (Yang H. et al., 2014), consistent with the significant QTN between the levels of glutamic acid (a typical cationic amino acid) and *TraesCS5D01G031800* locus (**Figure 3C** and **Supplementary Table S4**). Our analysis also placed *TraesCS2B01G493000* and *TraesCS7B01G093200* close to *AtAAP1*, *AtAAP6*, and *AtAAP8* (Hirner et al., 2006; Lee et al., 2007; Santiago and Tegeder, 2016) within clade I, strongly supporting the annotation of these candidates as AAPs in wheat (**Figure 3C**). Moreover, the high sequence identities between *TraesCS3B01G253600* and *AtGAT1* (63% at the amino acid level, Meyer et al., 2006), *TraesCS4A01G287900* and *AtPTR2* (44% at the amino acid level, Dietrich et al., 2004) provide further evidence for these assignments (**Figure 3C**).

### Functional Identification of Candidate Genes

Although experimental validation of all candidate genes disclosed by our mGWAS analyses is beyond the scope of a single study, we nevertheless tried to show that such confirmation is possible. For this purpose, we further characterized one candidate gene and provided novel biochemical insight into the FAA biosynthesis in wheat.

**FIGURE 4:**
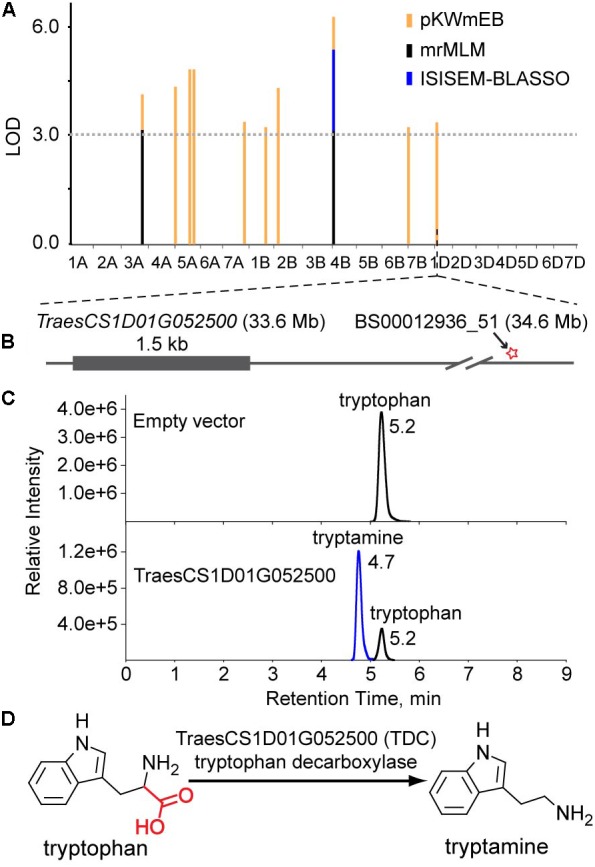
Functional identification of *TraesCS1D01G052500* *in vitro*. **(A)** The multi-locus GWAS results for the tryptamine level in different models. **(B)** Gene model of *TraesCS1D01G052500*. The filled gray box represents coding sequence, and the star represents the associated site. **(C)** LC-MS/MS chromatograms of *in vitro* enzyme assays showing the enzyme activity of recombinant TraesCS1D01G052500 (Down). Protein extract from *E. coli* containing empty vector were used as a negative control (Up). **(D)** The proposed pathway of tryptamine biosynthesis in wheat.

As mention above, the association between *TraesCS1D01G052500* and tryptamine levels suggests that TraesCS1D01G052500 is the decarboxylase that catalyzes the biosynthesis of tryptamine (**Figures 3B**, **4A,B**). To characterize the enzymatic properties of TraesCS1D01G052500, recombinant protein was expressed with an *N*-terminal glutathione *S*-transferase (GST) tag in *E. coli* BL-21 and the reaction product was confirmed by commercial standard with LC-MS (**Figure 4C**). An obvious TDC activity showed for tryptophan, and its activity was not inhibited by tyrosine, indicating a high level of substrate specificity toward tryptophan (**Supplementary Table S5**). We further investigated the enzyme kinetics of TraesCS1D01G052500 and its rice homologs (OsTDC1 and OsTDC3), all of them displayed similar *K*_cat_ values for tryptophan (**Supplementary Table S5**), suggesting that the three proteins have similar TDC activities. Based on these results, we functionally identified TraesCS1D01G052500 as a decarboxylase that catalyzes the biosynthesis of tryptamine from tryptophan in wheat (**Figure 4D**), which further confirmed the correctness of our GWAS results and the candidate gene assignment.

## Discussion

By coupling with the rapid development of LC-MS strategies, more accurate contents of metabolites can be obtained, and larger phenotypic variation can be observed (Chen et al., 2014). In this study, most of the FAAs varied widely across the association panel, such as proline with range of 30.4-fold (**Supplementary Table S2**), indicating the complexity of the biosynthetic processes of FAAs (**Figure 1**). The levels of lysine (an essential amino acid) have huge phenotypic variation, with a CV (%) of 77.2, implying the existence of a large number of alleles with high genetic diversity in the wheat germplasms (Liu Y. et al., 2017). Thus, identification of the favorable alleles and dissection of the genetic architecture underlying the levels of FAA is beneficial for improving the amino acid compositions in the future.

Dissecting the natural variation and the underlying genetic bases of metabolism is essential for the improvement of crop nutritional quality (Luo, 2015). Due to recent advances in both high-throughput metabolic profiling and sequencing technologies, mGWAS has been employed as a powerful strategy to reveal the genetic and biochemical basis of crop metabolism (Riedelsheimer et al., 2012; Wen et al., 2014; Matsuda et al., 2015). So far, most of these studies have been carried out on maize and rice. What’s more important, hundreds of significant loci were identified for various metabolites of nutritional importance, both of large effects and at high resolution, which facilitated the identification of the candidate genes (Luo, 2015). Advanced in developing the genomic toolbox (Jia et al., 2013; Ling et al., 2013; Avni et al., 2017), Matros et al. (2017) quantified 76 leaf metabolites from 135 winter wheat lines and identified several significant associations for six metabolic traits based on 17,372 SNP markers. This confirmed the potential of the mGWAS approach and provided the opportunity for a further understanding of metabolic diversity in wheat. In our study, we also mapped hundreds of QTNs for the levels of 20 FAAs in a wheat diverse association panel, however, most of them had very small effects, explaining the phenotypic variation with an average of 5.6% (**Supplementary Table S4**). Obviously, the limitations of mGWAS in wheat relate in part to the large size of the genome and in part to the limited availability of sets of genetic markers (Zhou et al., 2018), which leads to great difficulties to confirm the candidate genes. These constraints could be gradually complemented by applying new sequencing technologies and developing additional genomic markers (Liu Y. et al., 2017), and also, utilizing larger number of accessions and choosing more comprehensive choices of germplasms can enhance the power of mGWAS approaches, as demonstrated in rice and maize (Huang et al., 2012; Riedelsheimer et al., 2012).

As usual, variation of primary metabolites tends to be controlled by many small-effect loci. To increase the detection power of mGWAS, six multi-locus models were applied in this study. Totally, 328 significant QTNs were identified, however, only 4 SNP-trait associations were found with the single-locus model (MLM) at *P* ≤ 3.41 × 10^−6^ (**Table 1** and **Supplementary Table S3**). These results indicated the power of these multi-locus methods. Furthermore, the common QTNs appeared in different models confirming the credibility of these multi-locus GWAS approaches.

Based on these QTNs identified by the six multi-locus methodologies, candidates that have not been identified previously can be explored by searching for a protein or protein cluster that is biochemically related to the associated FAAs encoded at these loci. As a result, our mGWAS has allowed the assignment of 15 candidate genes underlying FAA levels (**Table 2**). The existing knowledge of plant FAA pathways, the high sequence identities between them and known functions in rice and *Arabidopsis* further confirmed these candidate genes. Notably, the validation of *TraesCS1D01G052500* was detected only by the pKWmEB model (**Figure 4**), further demonstrating the reliability and effectiveness of these multi-locus methods.

## Conclusion

In this study, a comprehensive GWAS of 20 FAA levels based on 14,646 SNPs in bread wheat was performed by six multi-locus models. Among 328 significant QTNs, 66 were detected by at least two models, and 155 QTNs appeared only in one model. Fifteen candidate genes were assigned to FAA biosynthesis, and one candidate gene was functionally identified *in vitro*. This study proved the power and reliability of multi-locus GWAS models in plant metabolome research and provided new insights into understanding FAA biosynthesis in wheat, which may facilitate metabolomics-based breeding for quality improvement.

## Author Contributions

WC, YH, and ZH conceived the project and supervised this study. YP, HL, and JC performed most of the experiments. TS, DS, and CZ participated in preparation of the materials. WC and YP analyzed the data. WC wrote the paper. All the authors discussed the results and commented on the manuscript.

## Conflict of Interest Statement

The authors declare that the research was conducted in the absence of any commercial or financial relationships that could be construed as a potential conflict of interest.

## Footnotes

1 http://www.capitalbiotech.com/

2 http://www.wheatgenome.org/

3 www.anpel.com.cn/

4 http://www.ncbi.nlm.nih.gov/
